# Quality Improvement Project: Lithium Monitoring in an Older Adult Community Mental Health Team

**DOI:** 10.1192/bjo.2024.402

**Published:** 2024-08-01

**Authors:** Aoife Nechowska, Rebecca Holman

**Affiliations:** Avon and Wiltshire Mental Health Partnership NHS Trust, Bristol, United Kingdom

## Abstract

**Aims:**

The aim of this project is to improve the monitoring of patients on lithium under the South Gloucestershire Later Life Community Mental Health Team and to clarify the process for this monitoring with the aim of improving patient care and safety. We aim to try to achieve 100% compliance with agreed standards based on NICE and Trust guidelines.

**Methods:**

Following a meeting with team medics we agreed a series of nine standards derived from local and national guidelines. We then used a locally held database of patients on the later life CMHT caseload on lithium therapy to identify our sample and devised a simple audit tool to collate the information. We used Rio electronic health records and ICE blood results to obtain baseline data from June 2022 to December 2022.

We used the plan-do-study-act (PDSA) cycle model for quality improvement. Following analysis of the baseline data, we planned and implemented key changes of the physical health nursing team taking over investigations from primary care and utilising a bespoke database. We also completed an education session for staff. Following these changes, data was collected and analysed from June until November 2023. From the analysis of these results, a further change was planned for PDSA cycle 2 and further data collection is planned.

**Results:**

Results from baseline data showed that six out of eight standards had compliance of < 60%, which included the time-sensitive investigations such as lithium levels every 3 months; kidney function tests every 3–6 months; calcium level every 6 months. Weight/BMI monitoring and documentation of side effects also had poor results. Average compliance across all standards was 57%.

Following the agreed steps to improve compliance, PDSA cycle 1 results showed improvement across the board, with average compliance increasing to 94%. Time-sensitive investigations now had 100% compliance (lithium level, kidney function, calcium level). Areas for improvement remain, namely in weight/BMI monitoring every 6 months and clear action plans for results falling out of range being clearly documented in patient notes.

**Conclusion:**

By working closely with the physical health nursing team to devise a bespoke local database of information and taking over the investigations from primary care, we have shown an improvement across all standards, therefore improving the quality of care and patient safety.

